# Older HIV-infected individuals present late and have a higher mortality: Brighton, UK cohort study

**DOI:** 10.1186/1471-2458-13-397

**Published:** 2013-04-26

**Authors:** Collins C Iwuji, Duncan Churchill, Yvonne Gilleece, Helen A Weiss, Martin Fisher

**Affiliations:** 1 Lawson Unit, Department of HIV/Genitourinary Medicine, Brighton and Sussex University Hospitals NHS Trust, Royal Sussex County Hospital, Eastern Road, Brighton BN2 5BE, United Kingdom; 2 Africa Centre for Health and Population Studies, University of KwaZulu-Natal, P.O. Box 198, Mtubatuba 3935, South Africa; 3 MRC Tropical Epidemiology Unit, London School of Hygiene & Tropical Medicine, London, WC1E 7HT, UK

**Keywords:** HIV, Late presentation, Older adults, Mortality, CD4 cell count

## Abstract

**Background:**

Initiating therapy with a low CD4 cell count is associated with a substantially greater risk of disease progression and death than earlier initiation. We examined factors associated with late presentation of HIV using the new European consensus definition (CD4 cell count <350 cells/mm^3^) and mortality.

**Methods:**

Patients newly diagnosed with HIV infection at a UK clinic were recruited from January 1996 to May 2010. Factors associated with late presentation were assessed using logistic regression. Factors associated with mortality rates were analysed using Poisson regression.

**Results:**

Of the 1536 included in the analysis, 86% were male and 10% were aged 50 years and older. Half the cohort (49%) had a CD4 cell count below 350 cells/mm^3^ at presentation (“late presentation”). The frequency of late presentation was highest in those aged 50 years or older and remained unchanged over time (64.3% in 1996-1998 and 65.4% in 2008-2010). In contrast, among those aged less than 50 years, the proportion with late presentation decreased over time (57.1% in 1996-1998 and 38.5% in 2008-2010). Other factors associated with late presentation were African ethnicity and being a male heterosexual.

The mortality rate was 15.47/1000 person-years (pyrs) (95%-CI: 13.00-18.41). When compared with younger adults, older individuals had a higher mortality, after adjusting for confounders (rate ratio (RR) = 2.87; 95%-CI: 1.88-4.40).

**Conclusions:**

Older adults were more likely to present late and had a higher mortality. Initiatives to expand HIV testing in clinical and community setting should not neglect individuals aged over 50.

## Background

Data from the Health Protection Agency (HPA) show that about one-quarter of people with HIV infection in the UK remain undiagnosed and 50% of adults diagnosed in 2010 had a CD4 cell count below 350/mm^3^ at diagnosis [1]. Initiating therapy with a CD4 cell count below 200 cells/mm^3^ is associated with a substantially greater risk of disease progression and death than earlier initiation [2]. Similarly, individuals entering the Strategies for the Management of Antiretroviral Therapy (SMART) study who were either treatment-naive or who had not been on therapy for the previous 6 months, and who deferred treatment until their CD4 cell count was below 250 cells/mm^3^ had a more than 4 fold higher risk of opportunistic infections and serious non-AIDS clinical events compared to patients who started treatment with CD4 cell count above 350 cells/mm^3^[3]. The SMART study [4] and other cohort studies [5,6] have shown that there is a continual gradient of increased risk of both death and disease progression associated with lower CD4 cell counts and no specific clear threshold at which risk increases.

The most frequent definition of late presentation for treatment is a CD4 cell count below 200 cells/mm^3^, although other thresholds have ranged from 50 to 350 cells/mm^3^[1,7-18]. This has sometimes been combined with whether an individual presented with an AIDS-defining diagnosis [19,20]. However, current UK guidelines recommend initiation of antiretroviral therapy (ART) in all patients with a CD4 cell count of less than 350 cells/mm^3^[21] and the European consensus is now that late presentation for care refers to persons presenting with CD4 cell count below 350 cells/mm^3^ or those presenting with an AIDS-defining event regardless of CD4 count [20,22]. Two recent studies have reported on late presentation using this European consensus definition. In one of these studies, 73% of CD4 cell count at diagnosis were missing and had to be imputed [23] while the other study examined late presentation over a shorter period of time compared to our study [24].

In this study, we examined factors associated with late presentation (CD4 cell count < 350 cells/mm^3^), and mortality in a large UK cohort comprising predominantly of men who have sex with men (MSM) over a 14-year period.

## Methods

### Study participants

**Figure 1:**
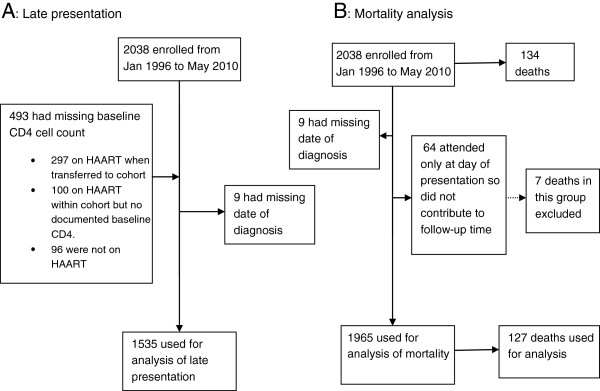
Cohort construction.

2038 patients aged over 18 years attended the sexual health clinic in Brighton between 1 January 1996 and 31 May 2010. Excluded from the analyses for late presentation were 493 (24.2%) patients with no documented baseline CD4 cell count for reasons stated in Figure 1A and a further 9 patients who had no documented date of HIV diagnosis, leaving 1536 (75.4%) patients with a documented CD4 cell count within 3 months of diagnosis eligible for this analysis.

For the analysis on mortality, 64 (3.5%) patients only attended once and did not contribute to follow-up time, while the dates of HIV diagnosis were missing for 9 patients leaving 1965 (96.4%) patients eligible for this analysis (Figure 1B). Ethical approval was not sought for this study as we utilized non-identifiable information previously collected in the course of normal care. Written informed consent was not required for the same reason [25], however access to the clinic database was approved by the HIV/Genitourinary Medicine Protocol Review Panel at Brighton and Sussex University Hospitals NHS Trust.

### Study/Clinic procedures

Patients testing HIV positive that were either diagnosed within the clinic or referred from another facilty were followed up at 3 monthly intervals. Demographic information collected at first visit were age at HIV diagnosis, date of HIV diagnosis, ethnicity and HIV risk group. The clinic database also had information on whether patients had been previously diagnosed elsewhere with HIV or not. This information ensured that true baseline CD4 cell counts were used for the analyses presented.

Patients initiating ART attended at 2, 6 and 10 weeks for blood tests and saw a doctor at 4, 8 and 12 weeks and 3 monthly subsequently after full suppression of the HIV RNA viral load.

CD4 cell count and HIV viral load within 90 days of HIV diagnosis were documented and subsequently measured at 3 monthly intervals in stable patients or more frequently at the discretion of their physician. The causes of death were entered into the clinic database from death certificates. Causes of death were ascertained for patients who died outside a healthcare facility following a coroner post-mortem examination and this was also entered into the clinic database when the information became available.

### Statistical analyses

To examine the proportion that presented late by calendar time stratified by age, calendar period of diagnosis was categorized as 1996-2001, 2002-2005 and 2006-2010 so that there were adequate numbers in each group for statistical analyses. Mean and standard deviations were reported for normally distributed variables, and median and inter-quartile ranges were used for skewed distributions. Frequencies and percentages were reported for categorical exposure variables.

Factors associated with late presentation were assessed using odds ratios (ORs) estimated using logistic regression. The likelihood ratio test statistic was used to estimate the *p*-values for association in the multivariable logistic regression model.

We also examined for the median time to initiation of HAART for the two age groups stratified by CD4 cell count at presentation using the Kaplan-Meier approach. For this analysis, patients were censored at the time started HAART for those on treatment and time last seen for those who remained untreated at the end of follow-up.

To estimate mortality rates, participants were censored at the earliest of: death, date last seen, or end of follow-up (31 May 2010). Poisson regression modelling was used to estimate survival probability. Patients who attended at least once following the enrollment visit contributed person-years (pyr) for the analyses. Age was the primary exposure when fitting the multivariable Poisson regression model. This was adjusted for a priori confounders such as ethnicity and HIV risk group irrespective of whether they acted as confounders in this study or not. All analyses were carried out using STATA 11.0 (StataCorp, College Station, TX, USA).

## Results

### Characteristics of study patients

Of the 2038 patients enrolled with HIV infection between January 1996 and May 2010, 1536 (75.4%) patients had documented CD4 cell counts within 3 months of diagnosis and formed the basis of the analysis on late presentation. Of the 1536 patients, 1316 (86%) were male and 151 (9.8%) were aged 50 years and older when diagnosed with HIV infection. The median age at HIV diagnosis was 33.1 years (IQR 27.8-38.7) and 54.8 years (IQR 52.0-60.0) respectively, for those aged <50 and ≥50 years at diagnosis and 34.2 years (IQR 28.2-41.5) for the whole cohort.

The majority of the cohort were Caucasian (79.5%) and MSM (73.8%). Of the 239 participants of African-Caribbean ethnicity, 90% acquired their HIV through heterosexual intercourse while 87% of Caucasians acquired their HIV through homosexual intercourse. There was no difference in the age and sex distributions of patients with missing CD4 count at presentation (*p* = 0.74 and 0.41 respectively). Patients with missing information on CD4 count were significantly more often heterosexual and of African ethnicity (*p* = 0.007 and 0.008 respectively).

### Factors associated with late presentation

Approximately half (49%) the cohort presented late with a CD4 count less than 350 cells/mm^3^ at HIV diagnosis and 23% (359/1536) presented with a CD4 count less than 200 cells/mm^3^. The median CD4 cell count for late and early presenters was 211 cells/mm^3^ (IQR 83-289) and 527 cells/mm^3^ (IQR 423-659) with median ages of 35.9 years (IQR 29.7- 43.6) and 32.9 years (IQR 27.5- 39.0) respectively.

On univariable analyses, late presentation was associated with older age, being of non-Caucasian origin and being either a female or male heterosexual (Table 1). On multivariable analyses, older age (OR = 2.18; 95%CI: 1.52-3.12; Table 1), male heterosexual, ethnicity (OR = 2.64, 95%CI 1.75-3.97 for African vs Caucasian) and earlier calendar period (OR = 0.62, 95%CI 0.48-0.81 for 2006-2010 vs 1996-2001) remained significantly independently associated with late presentation.

**Table 1:** Univariable and multivariable logistic regression of the association between age at diagnosis and other factors with late presentation

**N** = **1536**	**Late presenters N **(%)	**Early presenters N **(%)	**Crude odd ratios **(**95**% **CI**)	***p***-**value**	**Multivariable odd ratios **(**95**% **CI**)	***p***-**value**
**Age**				<0.001		<0.001
<50	658(47.5)	727(52.5)	1		1	
>50	96(63.6)	55(36.4)	1.93(1.36-2.73)		2.18(1.52-3.12)	
**Ethnicity**				<0.0001		<0.0001
Caucasian	540 (44.2)	681(55.8)	1		1	
African/Caribbean	170(71.1)	69(28.9)	3.10(2.30-4.20)		2.64(1.75-3.97)	
Others	35(54.7)	29(45.3)	1.52(0.92-2.52)		1.57(0.93-2.64)	
Unknown	9(75.0)	3(25.0)	3.78(1.02-14.04)		3.30(0.86-12.65)	
**Sex**/**Risk group**						
MSM	493(43.5)	641(56.5)	1	<0.0001	1	0.004
Female heterosexual	137(63.7)	78(36.3)	2.28(1.69-3.09)		1.27(0.85-1.90)	
Male heterosexual	117(68.8)	53(31.2)	2.87(2.03-4.05)		2.00(1.37-2.93)	
Others	7(41.2)	10(58.8)	0.91(0.34-2.41)		0.89(0.33-2.43)	
**Calendar period**				0.005		0.002
1996-2001	220(54.5)	181(45.1)	1		1	
2002-2005	282(49.8)	284(50.2)	0.82(0.63-1.06)		0.72(0.55-0.94)	
2006-2010	252(44.3)	317(55.7)	0.65(0.51-0.85)		0.62(0.48-0.81)	

MSM = men who have sex with men.

**Figure 2:**
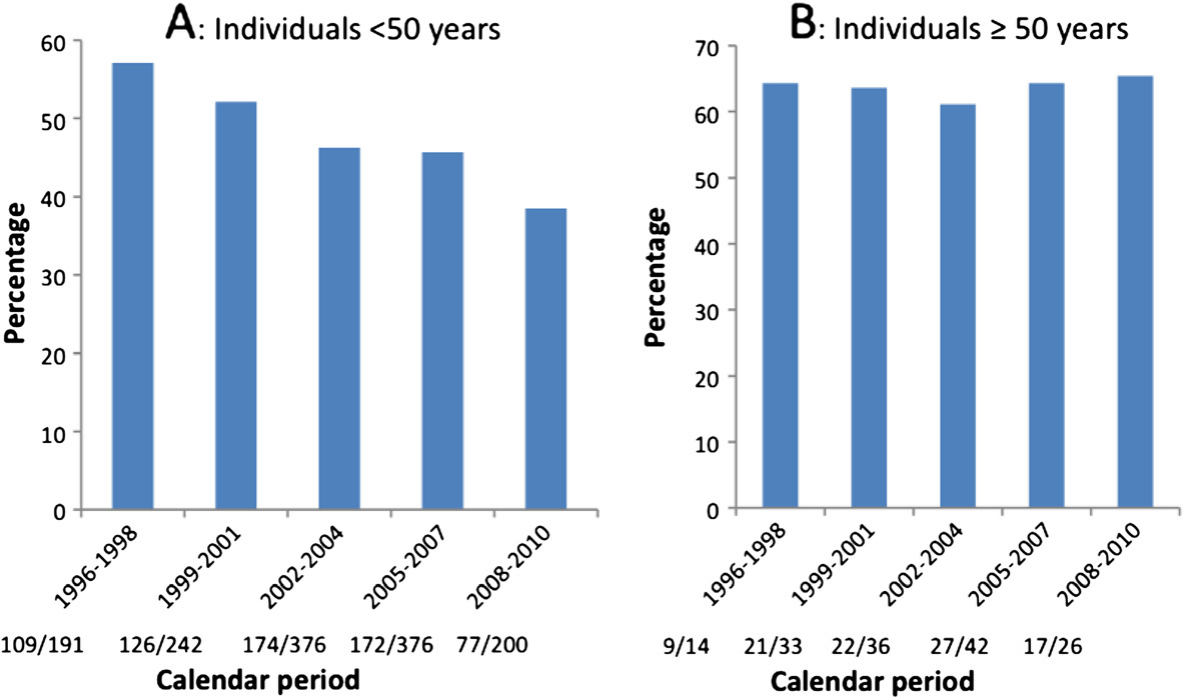
Proportion presenting late in different calendar periods according to age at diagnosis.

The proportion presenting late decreased from 57.1% among those younger than 50 years in 1996-1998 to 38.5% in the 2008-2010 (Figure 2A; *p*-trend = 0.0001). In contrast, among those over the age of 50 years, the proportion of patients presenting late was constant over time (Figure 2B; *p*-trend = 0.89).

There was also evidence of a difference in trends of presenting late with sexual orientation. Among MSM, 51.3% presented late in 1996-2001 period, compared with 42.4% in 2002-2005 and 39.1% in the 2006-2010 (*p*-trend = 0.0012). The corresponding proportions among heterosexuals were 66.3%, 66.5% and 65.0% respectively (*p*-trend = 0.78).

**Figure 3:**
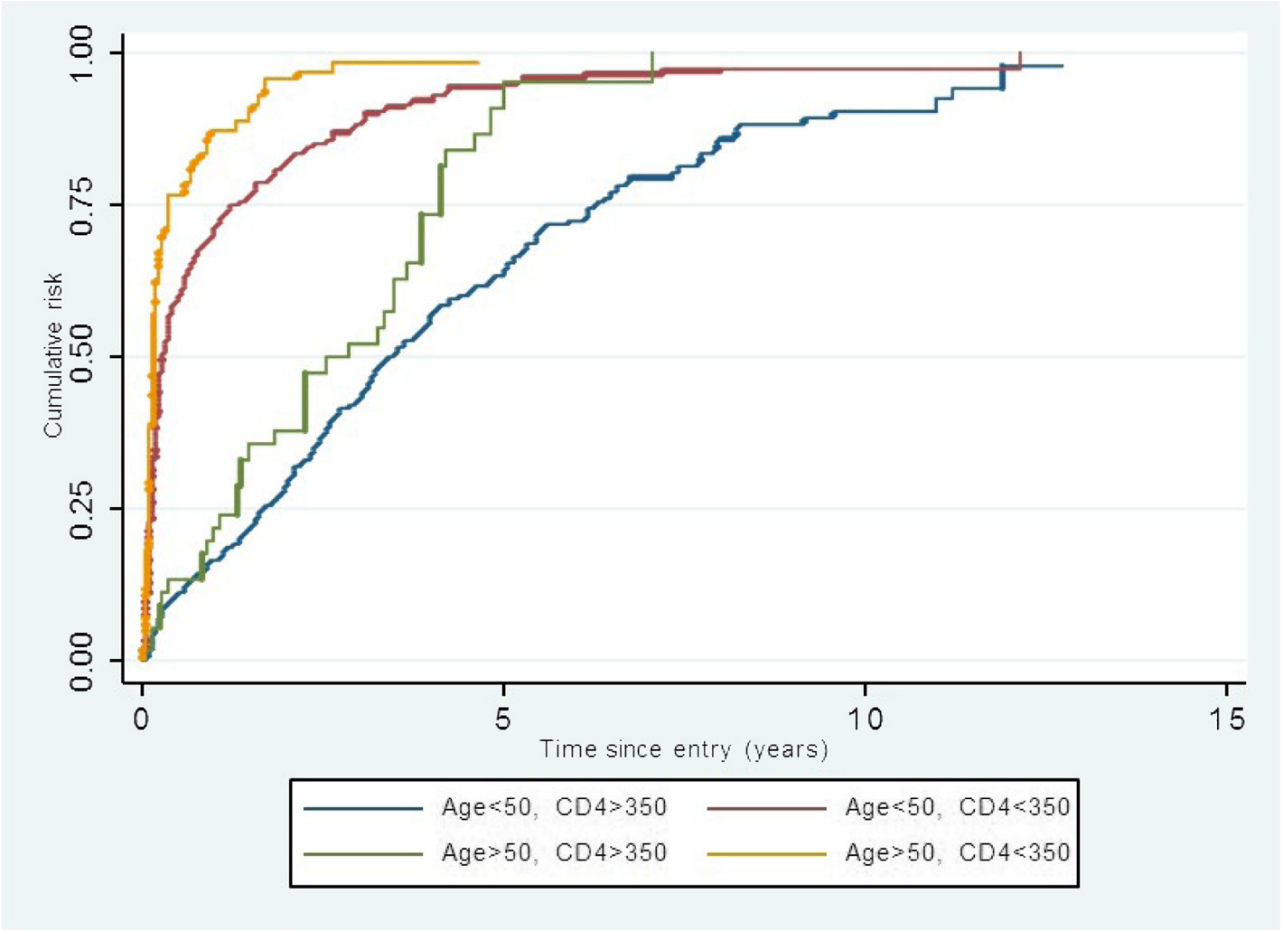
**Kaplan**-**Meier estimates of time to ART initiation by age and presentation CD4 groups.**

Median time to initiation of ART amongst those presenting late in those 50 years or older compared to those less than 50 years was 47 days (IQR 26.0- 131.0) and 98.0 days respectively (IQR 39.0 -476.0); *p* (log-rank test) = 0.001 whilst amongst those presenting early it is 1041 days (IQR 467-1491) and 1236 day respectively (IQR 607-2290); *p* (log-rank test) = 0.0001 (Figure 3).

### Mortality

Overall 127 deaths occurred in 1965 patients between Jan 1996 and May 2010; 26 and 54 deaths were in individuals presenting with CD4 cell count greater and less than 350 cells/mm^3^ respectively while 47 deaths occurred in individuals with unknown CD4 cell counts before death.

64/127 (50.4%) of deaths were AIDS related death, and were predominantly due to AIDS- related bacterial pneumonia and *pneumocystis jirovecii* pneumonia (PCP) as well as AIDS-related malignancies in both age groups. 34/127(26.8%) of deaths were non-AIDS related death, predominantly due to malignancies. The causes for the remaining deaths were unspecified.

Overall mortality rate in the cohort after 8210.30 person years (pyrs) of follow up was 15.47 per 1000 pyrs (95% CI: 13.00-18.41).

### Early mortality

53/127 (41.7%) of deaths occurred within the first year of follow up in cohort; 1/26(3.9%) in those presenting with CD4 count greater than 350 cells/mm^3^, 23/54(42.6%) in those with CD4 count less than 350 cells/mm^3^ and 29/47 (61.7%) in those with unknown CD4 count.

12/30 (40%) of deaths among individuals 50 years or older occurred in the first 12 months of presentation. None of these 12 deaths occurred in individuals with CD4 count greater than 350 cells/mm^3^. 4/12(33.3%) and 8/12(66.7%) were in individuals with CD4 count less than 350 cells/mm^3^ and unknown CD4 count respectively.

41/97(42.3%) of deaths among individuals less than 50 years occurred in the first 12 months of presentation. Only 1 of these early deaths occurred in an individual with CD4 cell count greater than 350 cells/mm^3^. 19/41(46.3%) and 21/41(51.2%) of these deaths were in individuals with CD4 count less than 350 cells/mm^3^ and Unknown CD4 count respectively.

### Factors associated with mortality

Table 2 shows factors associated with mortality in the cohort.

**Table 2:** Impact of age at diagnosis and late presentation with HIV on mortality

**N = 1965**	**Rate**/**1000 pyrs **(**95**% **CI**)	**Univariable rate ratio **(**95**% **CI**)	***p***-**value**	**Multivariable rate ratio **(**95**% **CI**)	***p***-**value**
**Age at HIV diagnosis **(years)			<0.0001		<0.0001
<50	13.16(10.79-16.06)	1		1	
>50	35.65(24.92-50.98)	2.70(1.80-4.08)		2.88(1.88-4.40)	
**CD4 count at diagnosis **Cells/mm^3^			<0.0001		<0.0001
>350	7.59(5.16-11.14)	1		1	
<350	16.65(12.75-21.74)	2.19(1.37-3.50)		2.99(1.80-4.95)	
Unknown	30.53(22.94-40.63)	4.02(2.49-6.50)		6.60(3.86-11.28)	
**Calendar period**			<0.0001		<0.0001
1996-2001	42.61(31.81-57.06)	1		1	
2002-2005	12.91(9.13-18.26)	0.30(0.19-0.48)		0.37(0.23-0.58)	
2006-2010	10.69(8.10-14.11)	0.25(0.17-0.38)		0.31(0.21-0.47)	
**Sex**/**HIV risk group**			0.06		0.43
MSM	14.20(11.53-17.50)	1		1	
Female heterosexual	12.73(7.39-21.93)	0.90(0.50-1.61)		0.90(0.46-1.78)	
Male heterosexual	26.16(17.54-39.03)	1.84(1.17-2.89)		1.45(0.90-2.35)	
Others	26.16(6.54-104.60)	1.84(0.45-7.48)		1.26(0.31-5.14)	
**Ethnicity**			0.09		0.04
Caucasian	15.21(12.56-18.41)	1		1	
African/Caribbean	12.53(7.28-21.58)	0.82(0.46-1.47)		1.05(0.53-2.10)	
Others	33.55(17.46-64.49)	2.21(1.12-4.36)		2.78(1.37-5.62)	
**ART status**			0.01		<0.0001
Not on ART	20.81(15.81-27.38)	1		1	
On ART	13.20(10.54-16.52)	0.63(0.44-0.90)		0.33(0.22-0.50)	

On Univariable analysis, individuals who were 50 years or older at HIV presentation had a higher mortality compared to younger adults-HR 2.70 (95% CI: 1.80-4.08). Being diagnosed with a CD4 count <350 cells/mm^3^ was associated with a doubling of mortality while ART was associated with 37% reduction in mortality.

On multivariable analysis, older age, earlier calendar period, and lower or unknown CD4 count were significantly associated with increased mortality. ART was also associated with a more marked reduction in mortality after adjustments. The reduction in mortality on ART was similar for the group who had been on it for less than 12 months with that of individuals who had been on it for longer, hence only the combined results for being on ART was shown. The decrease in mortality in the later calendar periods was further explored because of the change in UK guidelines in 2008 recommending the initiation of treatment when CD4 <350 cells/mm^3^. Prior to this ART was initiated at CD4 <200 cells/mm^3^. We observed that there was no difference in the adjusted decrease in mortality in the 3-year period preceding the change in guidelines and the 2-year period following this, both periods showing an 80% decrease in mortality (full results not shown).

No statistical interaction was detected between age at presentation and late presentation on mortality (*p*-interaction = 0.67).

## Discussion

In our study, 49% of those diagnosed with HIV were late presenters according to the new consensus definition for late presentation and nearly one-quarter presented with advanced HIV infection. The frequency of late presentation is similar to that recently reported from Germany and New Zealand of 49.5% and 50% respectively using the new threshold of CD4 <350 [23,24]. In this UK cohort, factors associated with late presentation were being older than 50 years, male heterosexual, African ethnicity and being diagnosed in the earlier calendar periods. The trend suggests HIV infection is increasingly being diagnosed early in the whole cohort and this was largely due to increased HIV testing in men who have sex with men as described in another cohort [26] and in those less than 50 years of age. The risk factors identified in this study corroborate findings from similar studies on late diagnosis [10,11,14,18,27].

We showed a progressive improvement in earlier diagnosis with time in those diagnosed under the age of 50 years but strikingly the likelihood of late presentation in those over 50 years of age remained constant over this 14-year period. The association of older age with late presentation of HIV has also been identified in other studies [11,13,14,19,28] that used other CD4 cell count thresholds for late presentation. However we know that individuals older than 50 years could have lower CD4 cell counts as a result of the normal ageing process even in the absence of HIV infection, so would be more likely to be considered “late presenters” when they acquire HIV infection.

A study from our group has shown that individuals older than 60 years were less likely to be offered an HIV test even in situations where an opt-out approach was adopted [29].

The failure of physicians to consider the possibility of HIV infections in these patients and confusion between symptoms of opportunistic infections and those of common co-morbid conditions associated with ageing further delays HIV diagnosis [30-32].

The association of African ethnicity with late presentation is consistent with other studies [11,14] and may reflect the fact that these groups of individuals are often marginalized and hard to reach. This may be due to lack of knowledge about HIV infection or reduced access to medical services [33]. Nearly all the Africans in our study acquired HIV infection through heterosexual intercourse, which could be due to sexual relations within or outside of the UK. A recent HPA report states that an increasing proportion of heterosexual transmission are occurring within the UK [34].

Trends suggest that early diagnosis of HIV infection is improving in the whole cohort. From 1996-2001, 55% of patients were diagnosed late using the current CD4 threshold, but this had dropped to 44% in the 2006-2010 calendar periods. This still represents a high burden of late presentation. Some studies have shown that many late presenters had suffered an HIV related illness, which had been missed in an earlier contact with a healthcare giver [9,35].

Being diagnosed with HIV with a CD4 cell count <350 and being older than 50 years of age were independently associated with increased mortality.

Late presentation in our cohort is associated with a tripling of mortality. This is the first study reporting a tripling of mortality with the higher CD4 count threshold for late presentation.

The association of late presentation with increased mortality has also been described in other studies both in the UK [18,36,37] and in other countries such as Spain [38], the Netherlands [39] and France [40]. These studies differ from ours in that they have used different CD4 cell count thresholds for defining late presentation most commonly a CD4 count less than 200 cells/mm^3^. Individuals diagnosed late are usually more likely to present with multiple HIV-related complications as well as have a higher risk of immune reconstitution inflammatory syndrome (IRIS), with a likely associated increase in mortality.

The strengths of this study lie in the utilization of the new European consensus definition for late presentation as this would facilitate regional comparisons within Europe and allow investigation of temporal trends after interventions [22], and the duration of the study period (14 years) which allowed a robust estimate of trends as well as large sample size. The study however has some limitations. The age at seroconversion was missing for the majority of the cohort; hence age at cohort entry (presentation) has been used. Age at entry is the sum of age at seroconversion and time since seroconversion [41,42], both of which are strong predictors of disease progression and death. In our study it was not possible to separate their two effects without knowledge of the date of seroconversion. We did not separate the effect of background mortality which is higher in older individuals from that associated with the additional effect of HIV infection. However, in individuals within the same age group, those presenting with lower CD4 cell count had a higher short-term mortality and this was more likely to be attributable to the effect of HIV itself rather than the effect of ageing of the cohort. In the author’s experience, some individuals with “missing” CD4 count at presentation (significantly more African and heterosexual risk group in this cohort) were those admitted through the emergency department in Brighton because of presentation with advanced HIV. Their CD4 counts, although in the hospital system did not always follow through to the clinic database which is different. We showed that these individuals have a higher mortality (unknown CD4 category), hence mortality would have been underestimated in those with CD4 <350 cells/mm^3^. Some deaths were coded as unspecified especially in those who died outside healthcare facilities as further information was lacking. This could have resulted in misclassification of some causes of deaths. Finally, the majority of the patients in this cohort were Caucasian MSM, hence the findings cannot be generalised to a cohort that is diverse in both ethnicity and sexual orientation; however this does not undermine the study validity.

As a result of the high mortality associated with late diagnosis, strategies must be put in place to encourage HIV testing. The CDC recommend routine testing in all healthcare settings for patients aged 13–64 years, unless the local HIV prevalence is known to be less than 0.1% [43].

In addition to recommending routine testing in genitourinary medicine and antenatal clinics, 2008 UK guidelines for HIV testing also advocate that tests should be offered to all adults registering in general practice and to all general medical admissions patients in areas where diagnosed HIV prevalence is greater than 2 per 1,000 among 15 to 59 year olds [44]. The UK guidelines have the advantage of not having any age restrictions but restricting HIV testing to only general medical admissions in hospitals within areas of high prevalence has been shown to miss the majority of HIV infections in one study [29].

HIV testing should take an ‘opt-out’ approach. Higher rates of HIV testing are achieved with such ‘opt-out’ approaches than opt-in approaches [45,46]. Identifying high-risk groups who continually refuse an HIV test and addressing the reasons for this could also encourage earlier diagnosis. One of the mains reasons for refusing a test is the stigma associated with HIV infection. To overcome the issue of stigma, we need to avoid the approach of offering an HIV test to only individuals perceived to be at high risk and ‘normalize’ the test by offering it to everyone alongside a battery of other routine investigations. Early HIV diagnosis could result in reduction of onward transmission as individuals aware of their HIV infection would be more likely to modify their sexual behaviours [47]. Furthermore, the use of HAART can also reduce an individual’s infectiousness by reducing plasma and genital HIV viral loads [48,49]. The findings of this study re-enforce the need for a universal HIV testing policy. General practitioners and hospital physicians should always be aware of the possibility of HIV infection in their patients irrespective of their age and there should be a policy that enables HIV test to be offered routinely alongside other investigations.

## Conclusion

This paper showed that HIV late presentation is very common and does not appear to be improving in older adults. This impacts individual health as documented by increased mortality in this study. The consequent increase in hospitalizations also results in increased health care costs. Most HIV transmissions are from individuals unaware of their status.

Prompt HIV diagnosis could be achieved through a policy that enables HIV test to be offered routinely alongside other investigations.

## Competing interests

All authors declare there are no competing interests that are relevant to this article.

## Authors’ contributions

CI conceived the study and participated in the design, analysis and draft of the manuscript. DC made substantial contributions to the study design and draft of the manuscript. YG contributed to the design and draft of the manuscript. HW contributed to the statistical analysis and draft of the manuscript. MF participated in the design, analysis, coordination and draft of the manuscript. All authors read and approved the final manuscript.

## Pre-publication history

The pre-publication history for this paper can be accessed here:

http://www.biomedcentral.com/1471-2458/13/397/prepub
